# Editorial: Soil fungal biodiversity for plant and soil health, volume II

**DOI:** 10.3389/fmicb.2023.1170312

**Published:** 2023-03-21

**Authors:** Magdalena Frąc, Małgorzata Jędryczka, Emilia Silja Hannula

**Affiliations:** ^1^Institute of Agrophysics, Polish Academy of Sciences, Lublin, Poland; ^2^Institute of Plant Genetics, Polish Academy of Sciences, Poznań, Poland; ^3^Institute of Environmental Sciences, Leiden University, Leiden, Netherlands

**Keywords:** soil-borne fungi, food-borne fungi, detection of fungi, antifungal agents, plant health, microbiome

Fungi represent a large portion of the biodiversity on Earth and they are key players in soils where they provide numerous ecosystem functions. Soil fungi play important ecological roles influencing plant health as symbionts, pathogens and decomposers. Soil fungal biodiversity is increasingly recognized as providing benefits to soil health as they participate in numerous ecosystem processes. Continued research on the identity, abundance and distribution of soil fungi, their various roles in soil microbiome community are thus fundamental to better understand all dimensions of fungal biodiversity, their impact on plant health as well as the prevention of diseases.

This Research Topic aimed at collecting contributions that provide taxonomic, physiological and ecological characterizations of soil fungal communities that will aid to the understanding of their biology, interrelationships and the mechanisms that underpin the various ecosystem functions they provide in the soil. The volume focuses at environmental mycology and reports sensitive, accurate and fast methods for the detection, identification and distribution of fungi. We were interested in metagenomic, metatranscriptomic and metabolomic approaches, as they increasingly reveal the impact of fungal biodiversity on soil and plant health.

Monoculture farming is a fascinating testing ground for studies of soil mycobiome. The use of various cultivars, application of agronomic methods, pesticides, fertilizers and biological compounds at different stages of plant development cause changes in the structure and dynamics of soil microbiome (Frąc et al.). They can be rapid or subtle, depending on the applied factor. In this volume of Frontiers in Microbiology we have concentrated on mycobiome, which is much less popular in soil microbiome studies than bacteria. Wolińska et al. focused their research on identification of the core fungal mycobiome and found fungi linked to the sensitivity and resistance to long-term monocultures of maize, which is one of the most popular crop plants grown in monocultures worldwide. The study clearly demonstrated mycobiome changes depending on the seasons and agricultural practices. Even short breaks in the monoculture, caused by intercropping, greatly increased soil mycobiome biodiversity. Similarly, the soil mycobiome dramatically changed after the use of cover crops, in the soil of continuous cucumber cultivation under plastic shed systems (Ali et al.), mainly due to the increase of beneficial fungi. The study indicated positive sides of cover crops in creating plant-protective mycobiome in the soil.

Soil microbes play numerous functions in the ecosystem and interact with plants improving their growth, development, fitness and production (Frąc et al.). However, in most plants there are only fragmentary results concerning the effects of planting patterns on the fungal community structure in the rhizospheric soil. The results obtained by Cai et al. indicated that fertilization alters the fungal community structure in soil rhizosphere of cassava, increase the potentially beneficial fungi and decrease the pathogenic species. Moreover, the results showed that both full-dose and reduced fertilization significantly increased plant yield. The authors paid attention to the need of maintaining the stability of ecological niches in crop plant rhizosphere and they suggested to use specific microbial-based fertilizers. Jin et al. underlined that the decrease of chemical fertilizer application in combination with bio-organic amendments can be effective in reducing the overuse of mineral fertilizers in agriculture without the loss of soil fertility. Soil mycobiome was more responsive than bacterial microbiome to chemical fertilizer reduction combined with bio-organic amendments applied to lettuce. This fertilization strategy improved soil fertility, as well as plant yield and quality, giving valuable implications for vegetable safety, and environmental sustainability development.

Koch and Herr mined the global ITS sequence datasets for the presence of *Armillaria* species to gain better understanding of the global distribution of this important species-group popular in forest and agaric ecosystems. The authors showed that *Armillaria* genus is present almost everywhere but the highest diversity is found in Eastern Asia. ITS1 and ITS2 regions are similar in their ability to describe species richness and the results on species richness derived from ITS-based analyses are in line with morphological and reproductive behavior. This is truly showing the potential of using locally collected information to perform global assessment of distribution models as well as evaluation of temporal changes in species distribution e.g., due to climate change. In line with Frac et al., these global analyses are only possible when the same marker regions and methods are used across studies and when the data are shared in publicly available databases. The use of the same universal barcoding region such as ITS has made these sort of analyses possible for fungi (Schoch et al., 2012). However, the identification of species requires additional fragments (Frac et al.).

## Conclusions

Soil mycobiome (fungal microbiome) is essential, but still neglected, component of soil microbiome. Soil fungi are very important for agricultural, horticultural and forest ecosystems supporting functioning and environmental services to plant health, soil quality, fertility, and ecological stability. Soil functionality can be improved by managing the soil mycobiome. However, fungi can also cause plant diseases, decrease yield, and affect human health. Therefore, there is a need to study the possibility of different mycobiome-based solutions and tools for sustainable and regenerative agriculture, including prediction of plant disease occurrence and control of mycobiome shift in soils. Metagenomic, metatranscriptomic, and metaproteomic methods combined with bioinformatic analyses may help to protect crops in modern agriculture. Moreover, there is a necessity to monitor environmental effects of newly developed microbial-based solutions. Although the knowledge on soil fungal community and mycobiomes is increasing, there is still a big black box concerning the future aspects of its influence on the environment.

## Author contributions

MF, EH, and MJ wrote drafted, read, corrected, improved, revised, and accepted the last version of manuscript. All authors contributed to the article and approved the submitted version.
